# A network of RNA and protein interactions in Fronto Temporal Dementia

**DOI:** 10.3389/fnmol.2015.00009

**Published:** 2015-03-19

**Authors:** Francesca Fontana, Kavitha Siva, Michela A. Denti

**Affiliations:** ^1^Laboratory of RNA Biology and Biotechnology, Centre for Integrative Biology, University of TrentoTrento, Italy; ^2^CNR, Institute of NeurosciencePadua, Italy

**Keywords:** FTD, TDP-43, FUS, progranulin, tau, CHMP2B. C9ORF72

## Abstract

Frontotemporal dementia (FTD) is a neurodegenerative disorder characterized by degeneration of the fronto temporal lobes and abnormal protein inclusions. It exhibits a broad clinicopathological spectrum and has been linked to mutations in seven different genes. We will provide a picture, which connects the products of these genes, albeit diverse in nature and function, in a network. Despite the paucity of information available for some of these genes, we believe that RNA processing and post-transcriptional regulation of gene expression might constitute a common theme in the network. Recent studies have unraveled the role of mutations affecting the functions of RNA binding proteins and regulation of microRNAs. This review will combine all the recent findings on genes involved in the pathogenesis of FTD, highlighting the importance of a common network of interactions in order to study and decipher the heterogeneous clinical manifestations associated with FTD. This approach could be helpful for the research of potential therapeutic strategies.

## Frontotemporal dementia

Despite 90% of the human genome being transcribed to RNA, only 1.2% of genomic sequence is protein-coding, indicating that a huge proportion of non-coding RNAs (ncRNAs) are likely to participate in a number of physiological processes in cell types, including neurons (Lander et al., 2001; Birney et al., 2007; Wilhelm et al., 2008; Clark et al., 2011). The transcribed precursors of messenger RNAs (pre-mRNA) undergo splicing, such that the non-coding introns are removed and exons are combined variably to produce an RNA that would code for protein (Pandit et al., 2008). The pre-mRNAs undergoes alternative splicing producing mature messenger RNAs (mRNAs) which are then expressed in specific tissues and cell types in different stages of development. These mRNAs then associate with the ribosomal machinery to be translated into proteins in the cytoplasm. Non-coding RNAs (among which microRNAs and long non-coding RNAs), might regulate the translation of specific mRNAs, thereby representing a post-transcriptional mechanism exerting a fine-tuned control in the production of specific proteins.

**Figure 1:**
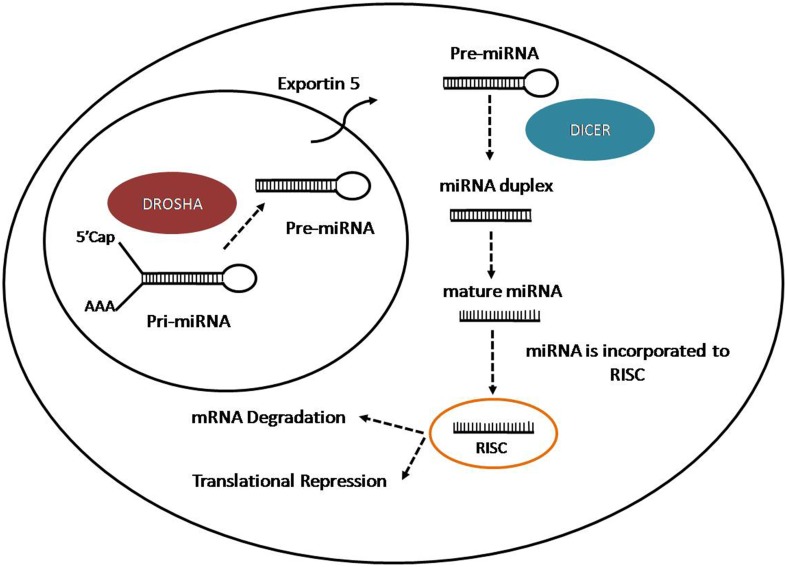
**Representation of miRNA biogenesis**.

microRNAs (miRNAs) are a group of small non-coding RNAs of 21–22 nt with important regulatory roles on the post-transcriptional expression of target mRNAs (Bartel, 2009; Ghildiyal and Zamore, 2009). MiRNAs are generating from longer transcripts of different lengths called primary transcripts (pri-miRNAs), usually transcribed by RNA polymerase II, from intragenic or intergenic DNA regions (Lee et al., 2004; Garzon et al., 2010). The pri-miRNAs are processed in the nucleus by the micro-processor complex, formed by an RNase III enzyme, Drosha, and its cofactor DiGeorge syndrome critical region in gene eight termed (DGCR8) (Lee et al., 2003). The process lead to the production of small hairpin structure of 70–100 nt called precursor miRNAs (pre-miRNAs). Pre-miRNAs are exported to the cytoplasm through Exportin 5 (Kim, 2004), where they are further processed by an RNase III nuclease, Dicer to produce RNA duplex (Bernstein et al., 2001; Grishok et al., 2001; Hutvagner et al., 2001). One strand is loaded on the RNA-Induced Silencing Complex (RISC) and associated with Argonaute-2 (Ago2) to interact with the target mRNA. The miRNA-RISC complex induces mRNA downregulation through two different ways: mRNA cleavage in case of perfect complementarity between miRNA and target mRNA or translation inhibition if there is an imperfect binding (Wahid et al., 2010) (Figure 1). In case of perfect complementarity, Ago2 is the protein involved in the cleavage of the target mRNA in humans (Liu et al., 2004). However, in animals, translational repression is the most frequent way of action for miRNAs (Huntzinger and Izaurralde, 2011; Pasquinelli, 2012), although the exact process is still unknown since is not clear if the repression occur at the initiation step or during the translation process (Wahid et al., 2010). Even the mechanisms for target regulation played by miRNAs are still unclear, the target mRNA could be repressed by the promotion of deadenylation, sequestration of miRNAs and target by stress granules and P-Bodies (Valencia-Sanchez et al., 2006), disruption of translation initiation or protein degradation caused by RISC after translation (Tang et al., 2008).

The enormous content of non-coding RNA (ncRNA) in the cell intrigues its role and function in the cells. LncRNAs are defined as transcripts longer than 200 nucleotides and lacking an appreciable open reading frame (usually less than 100 amino acids). They may be transcribed by RNA polymerase II (RNA Pol II) or RNA Pol III, and may undergo splicing or comprise of a single exon. In contrast to small ncRNAs, lncRNAs tend to be poorly conserved evolutionarily and regulate gene expression by diverse mechanisms that are not entirely understood. As a functionally diverse macromolecule, the biological roles of lncRNAs cannot be determined solely from their nucleotide sequence, secondary structures, or genomic locations (Ng et al., 2013).

Recent work has begun to elucidate the roles of some lncRNAs, such as architectural function in nuclear paraspeckles (Sunwoo et al., 2009; Souquere et al., 2010), transcriptional co-regulators (Feng et al., 2006; Bond et al., 2009), and as endogenous competing RNAs (ceRNAs) (Cesana et al., 2011; Tay et al., 2011). LncRNA expression is abundant in cells of the CNS (Mehler and Mattick, 2007; Mercer et al., 2008) and recent studies have suggested that lncRNAs play crucial roles in spatial-temporal control of gene expression in brain development (Mercer et al., 2008). They have also known to be involved in brain development, neural differentiation and maintenance, synaptic plasticity, cognitive function and memory, and in aging and neurodegenerative disorders (Wu et al., 2013b).

Though different mechanisms may play a role in causing neurodegenerative disorders, recent studies show increasing evidence of abnormalties in RNA processes, highlighting the possible putative role of RNA in neurodegeneration. An mRNA gain-of-toxic-function has been proposed for some neurodegenerative diseases (Osborne and Thornton, 2006; O'Rourke and Swanson, 2009; Todd and Paulson, 2010) whereas other neurodegenerative disorders are driven through altered or lost non-coding RNA, RNA splicing and RNA binding activities (Gallo et al., 2005; Cooper et al., 2009; Lagier-Tourenne et al., 2010).

Fronto temporal lobar degeneration (FTLD) is the most common cause of dementia after Alzheimer's disease. The clinicopathological spectrum of FTLD includes frontal and temporal variants of frontotemporal dementia (FTD), primary progressive aphasia, semantic dementia, Cortico-basal degeneration (CBD), progressive supranuclear palsy (PSP), progressive subcortical gliosis (PSG) and FTD with motor neuron disease (FTD–MND) (Bugiani, 2007). Moreover, despite Amyotrophic Lateral Sclerosis (ALS) and FTD being two different neurodegenerative disorders, they often share genetic, neuropathological and clinical characteristics; therefore they are considered part of the same spectrum of diseases (Ling et al., 2013). Frontotemporal dementia symptoms can also be present along with disabling muscle weakness and osteolytic bone lesions, in IBMPFD1 (Inclusion body myopathy with early-onset Paget disease with or without Frontotemporal dementia 1).

It is estimated that one in seven people in the US might develop a neurodegenerative disorder in their lifetime, with dementia being one of the leading causes of death in US (Thies and Bleiler, 2011). Though this broad spectrum of disorders has been studied based on protein aggregation and research has been focusing on protein functions and alterations, emerging avenues in research unravels the role of RNA and RNA processing in contributing to neurodegeneration (Belzil et al., 2013).

To date, FTD has been linked to mutations in seven different genes (*TARDBP, FUS, MAPT, GRN, VCP, CHMP2B, C9ORF72*).

Findings that showed the presence of ubiquitinated protein TDP-43 in sporadic cases of ALS with FTD further linked these two diseases (Arai et al., 2006; Neumann et al., 2006). Following these findings, mutations in the gene coding for the RNA binding protein TDP-43 were discovered in ALS cases (Kabashi et al., 2008; Sreedharan et al., 2008; Van Deerlin et al., 2008) and FTD cases (Borroni et al., 2009; Kovacs et al., 2009).

With the broadening knowledge on the impact of impaired RNA binding proteins in mediating the disease process, mutations in the fused in sarcoma/translocated in liposarcoma (*FUS/TLS*) gene were found to account for an additional 5% of familial ALS and few rare cases of FTD (Kwiatkowski et al., 2009; Vance et al., 2009).

TDP-43 and FUS share similar structural and functional properties with a likely role in multiple steps of RNA processing and they are both linked to RNA metabolism. The pathological accumulation of these proteins is observed in over 90% ALS and 50% FTD patients. These studies also highlight that errors in RNA processing might be enough to initiate the disease process.

*MAPT* mutations were observed in several FTD families with abnormally phosphorylated tau proteins being isolated from neuroectoderm cells of patients. Mutations present in the C terminal repeat domains lead to the inhability of abnormal tau protein to bind microtubules, thus leading to its instability and accumulation and causing neuronal degeneration (Bugiani, 2007). FTD with tau inclusions was characterized as a tauopathy and dubbed FTLD-tau.

However, a different class of patients were found to have had accumulated ubiquitin and ubiquitin-associated proteins (FTLD-U). Co-localization of abnormal proteins with ubiquitin in the nucleus and perikaryon of neuronal cells, indicated the involvement of proteasome dysfunction in the pathology. Analysis of significant genes on chromosome 17, close to the MAPT locus, led to the discovery of mutations in *GRN* (Baker et al., 2006; Cruts et al., 2006). GRN is known to be involved in the cell cycle control and motility.

Studies on an ALS/FTD-affected Scandinavian family (Morita et al., 2006) and on IBMPFD1 families suggested the possible role of mutations in chromosome 9 in FTD. The disorder was associated to mutations in *VCP*, encoding the valosin-containing protein essential for ubiquitin-mediated protein degradation (Watts et al., 2004; Johnson et al., 2010).

Other FTLD mutations are located on chromosome 3 (FTD-3), in the *CHMP2B* gene, which encodes for a protein involved in degradation of surface receptor proteins and formation of endocytic multivesicular bodies (Skibinski et al., 2005).

Another link between ALS and FTD are the large intronic hexanucleotide repeat expansions in the *C9ORF72* gene located on chromosome 9 found in ALS, FTD, or ALS/FTD cases (DeJesus-Hernandez et al., 2011; Renton et al., 2011; Gijselinck et al., 2012).

This review will focus on the single genes known to have implications in FTD and their altered functions in the diseased state. The ultimate aim is to explorepossible functional connections between these seven diverse genes and describe a network in which a possible common thread might be represented through RNA mediated processes.

## *TARDBP* (TDP 43)

Human TDP-43 was discovered in 1995 in a screen for transcriptional repressors of the trans-active response (TAR) DNA binding element of the HIV-1 virus, and thus the gene is named TARDNA Binding Protein (*TARDBP*) (Ou et al., 1995). *TARDBP* is composed of six exons and maps on chromosome 1p36.22.

The protein *TARDBP* produces is being labeled as TDP-43 due to its molecular weight of 43 KDa (Neumann et al., 2006). *TARDBP* is ubiquitously expressed in various human tissues (Table 1) including brain and spinal cord (Wang et al., 2008a). To date, 34 different TDP-43 mutations have been discovered in 131 different FTD and ALS families (Cruts et al., 2012). Pathogenic mutations observed in TDP-43 are highlighted in Table 2.

**Table 1:** **Protein localisation of different genes associated to FTD**.

**Gene**	**Genomic location**	**Protein**	**Tissue localization in the brain**	**Cell type**	**Subcellular localization**
*TARDBP*	Chromosome 1 p36.22	TDP-43	Cerebral cortex, hippocampus, lateral ventricle, cerebellum and spinal cord	Endothelial, neuronal, glial cells, neuropil and cell in granular and molecular layer, Purkinje cells	Nucleus and cytoplasm
*FUS*	Chromosome 16 p11.2	FUS	Cerebral cortex, hippocampus, lateral ventricle and cerebellum	Endothelial, neuronal, glial cells, neuropil and cell in granular and molecular layer, Purkinje cells	Nucleus and cytoplasm
*MAPT*	Chromosome 17 q21.3	Tau	Cerebral cortex, hippocampus, lateral ventricle and cerebellum	Neuronal, glial cells, neuropil and cell in granular and molecular layer, Purkinje cells	Cytoskeleton, cytoplasm, nucleus and plasma membrane
*GRN*	Chromosome 17 q21.31	Progranulin	Cerebral cortex, hippocampus, lateral ventricle and cerebellum	Neuronal, glial, endothelial cells and cell in granular layer	Vesicles, endoplasmic reticulum, golgi, extracellular space
*VCP*	Chromosome 9 p13.3	VCP	Cerebral cortex, hippocampus, lateral ventricle and cerebellum	Endothelial, neuronal, neuropil, glial cells and cell in granular and molecular layer, Purkinje cells	Endoplasmic reticulum, nucleus, cytoplasm
*CHMP2B*	Chromosome 3 p11.2	CHMP2B	Lateral ventricle	Neuronal cells	Cytosol, endosome, nucleus, mitochondria
*C9ORF72*	Chromosome 9 p21.2	C9ORF72	Cerebral cortex, hippocampus and lateral ventricle	Endothelial, neuropil, glial cells	Cytoplasm, nucleus, cytoskeleton

*The information provided are derived through integration of two different databases (http://www.genecards.org/ and http://www.proteinatlas.org/) and literature reported in the text*.

**Table 2:** **List of mutations in *TARDBP* and their characteristic phenotypes**.

**Subtypes of Dementia**	**Mutation**	**Change in amino acid**	**Type**	**References**
FTD	g.6142C>T	p.N12	Pathogenic	Luquin et al., 2009
FTD	g.9253C>T	A90V	Pathogenic	Sreedharan et al., 2008
FTD/PSP	g.14575A>G	K263E	Pathogenic	Kovacs et al., 2009
ALS/FTD	g.14588A>G	N267S	Pathogenic	Corrado et al., 2009
FTD	g.14671G>A	G295S	Pathogenic	Benajiba et al., 2009
FTD	g.14932G>A	A382T	Pathogenic	Chiò et al., 2010

*FTD, Frontotemporal Dementia; PSP, Progressive supranuclear palsy*.

*All the information reported in the table is derived from a cumulative study of the literature and the database: http://www.molgen.ua.ac.be/ADMutations/default.cfm?MT=0&ML=2&Page=FTD*.

### Structure

**Figure 2:**
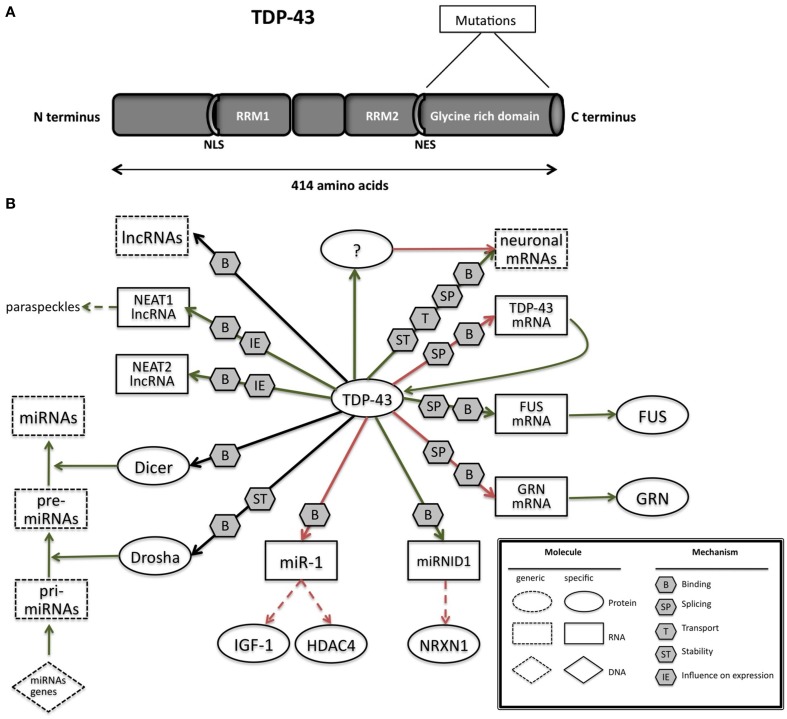
**(A)** TAR DNA-binding protein 43 (TDP-43) contains two RNA-recognition motifs (RRM1 and RRM2), a carboxy-terminal glycine-rich domain, a bipartite nuclear localization signal (NLS), and a nuclear export signal (NES). Mutations are mainly located in the glycine-rich domain. **(B)** The network of interactions of TDP-43 with proteins and RNAs. Green arrows indicate binding interactions or processes that result in activation or increased expression. Red arrows indicate binding interactions or processes that result in inhibition of activity or reduced expression. Black arrows indicate binding interactions or processes whose result can be either positive or negative. Dashed arrows indicated indirect processes. Symbols as in Legend. lncRNAs, long non-coding RNAs; IGF-1, insulin-like growth factor 1; HDAC4, histone deacetylase 4; NRXN1, neurexin 1; TDP-43, TAR DNA binding protein; FUS, fused in sarcoma; GRN, progranulin; NEAT1, nuclear-enriched autosomal transcript 1; NEAT2, nuclear-enriched autosomal transcript 1.

TDP-43 is a 414 amino acids protein (Figure 2A) containing two RNA recognition motifs (RRMs), a glycine-rich low sequence complexity prion-like domain (Wang et al., 2013). A nuclear localization signal motif (NLS) and a nuclear export signal motif (NES) allow TDP-43 to shuttle between the nucleus and the cytosol (Buratti and Baralle, 2001).

### Localization and function

Though TDP-43 expression is seen in the nucleus with low cytosolic localization (Ayala et al., 2005), there is a significant cytosolic TDP-43 expression especially in large motor neurons where TDP-43 has an additional role in mRNA transport as a neuronal activity responsive factor in dendrites thus promoting dendritic branching (Wang et al., 2008a; Barmada et al., 2010; Kabashi et al., 2010).

TDP-43 was found to be accumulated in cytoplasmic stress granules due to oxidative stress (Colombrita et al., 2009). Stress granules are aggregations, formed after cell insults such as oxidative stress or heat shock that temporarily store non-translating mRNAs, small ribosome subunits, RNA-binding proteins and translation initiation factors (Buchan and Parker, 2009). Formation of stress granules protects the cells, allowing a translational block and initiation of repair processes (Anderson and Kedersha, 2008).

Upregulation of nuclear TDP-43 has also been shown to provide protection to primary neurons against glutamate induced excitotoxicity (Zheng et al., 2012). These findings also suggest that TDP-43 regulates synaptic plasticity by governing the transport and splicing of synaptic mRNAs. In a recent review, Belzil and co-authors postulate that altered TDP-43 could lead to impaired hippocampal plasticity and render neurons more vulnerable to cellular stressors (Belzil et al., 2013).

TDP-43 is highly conserved from human to *C. elegans*, both in the RNA binding motifs and in the carboxy-terminal portion (Ayala et al., 2005). *In situ* hybridization studies showed that TDP-43 is expressed very early in the brain and spinal cord of zebrafish (Shankaran et al., 2008) suggesting that it plays an important role in nervous system development.

### Implications of RNA in pathogenesis

Many studies have linked TDP-43 to neurodegenerative disorders, including ALS and FTLD (Neumann et al., 2006; Lagier-Tourenne et al., 2010; Lee et al., 2012). Janssens and Van Broeckhoven (2013) have highlighted the increasing evidence of role of impaired RNA metabolism in TDP-43-driven neurodegeneration.

*TARDBP* primary transcript undergoes alternative splicing to produce eleven different mRNAs including the one encoding TDP-43. Seven of these are shorter transcripts which are generated through the seven different splicing reactions within exon 6 of *TARDBP* pre-mRNA using a combination of four different 5′ donor sites and four different 3′ acceptor sites (Wang et al., 2004a).

In few ALS cases a smaller TDP-43 isoform (~28 KDa) was observed additionally to the 43 kDa isoform, lacking exon 3 and a significant portion of exon 6-encoded amino acids (Strong et al., 2007). This smaller isoform lacks the carboxy-terminal portion of the protein and is thought to be associated with disease pathology (Neumann et al., 2006).

Converging lines of evidence in research suggest that TDP-43 regulates RNA in various ways (Figure 2B; Lee et al., 2012). The RRM1 domain of TDP-43 is critical for its binding to single-stranded RNA (Ou et al., 1995; Buratti and Baralle, 2001; Wang et al., 2004a; Ayala et al., 2005). TDP-43 preferentially binds UG repeats, but is also found to be associated with non-UG repeat sequences (Buratti and Baralle, 2001; Ayala et al., 2005; Polymenidou et al., 2011; Tollervey et al., 2011).

Pathological TDP-43 aggregates are ubiquitinated and phosphorylated. Under normal conditions, these forms are not readily detectable in brain tissues, thus making them disease-specific. Over-expression of full-length TDP-43 in a variety of transgenic animal models lead to the presence of phosphorylated TDP-43 aggregates similar to ALS and FTD cases (Wegorzewska et al., 2009; Shan et al., 2010; Stallings et al., 2010; Wils et al., 2010; Xu et al., 2010a). The phosphorylated form has a longer half-life than the non-phosphorylated form thus leading to accumulation of phosphorylated proteins. Despite the progress toward describing the full spectrum of TDP-43 pathology in human neurodegenerative diseases, the fundamental question of whether TDP-43 dysfunction mediates neuro-degeneration through gain of toxic function or a loss of normal function remains unanswered (Lee et al., 2012).

Upon depletion of TDP-43 from adult mouse brain with antisense oligonucleotides, levels of 601 mRNAs, including FUS, GRN and other transcripts involved in neurodegeneration, were altered, along with 965 varied splicing events. RNAs depleted by the reduction of TDP-43 were coded by genes with long introns (Polymenidou et al., 2011).

*In-vivo* searches for TDP-43 RNA targets in mouse (Polymenidou et al., 2011), human brain (Tollervey et al., 2011), rat cortical neurons (Sephton et al., 2011), a mouse NSC-34 cell line (Colombrita et al., 2012), and a human neuroblastoma cell line (Xiao et al., 2011) revealed that there are more than 6000 RNA targets which constitutes to about 30% of total transcriptome. TDP-43 was found to preferentially bind to introns (including deep intronic sites), 3′ untranslated regions (UTRs), and non-coding RNAs (Polymenidou et al., 2011; Tollervey et al., 2011), indicating a multifaceted role in RNA maturation. TDP-43 can influence splice site selection by binding to exon-intron junctions and intronic regions, mRNA stability and transport by binding on 3′UTRs. A substantial amount of mRNAs regulated by TDP-43 at splicing levels were involved in neuronal development or in neurological diseases (Tollervey et al., 2011). Additional data show that when TDP-43 is reduced the levels of several other mRNAs increase. As the affected mRNAs include more than 300 mRNAs without TDP-43 binding sites, these observation point toward an indirect mechanism (Polymenidou et al., 2011) of modulation.

i-CLIP experiments have also shown that TDP-43 binds to long ncRNAs (lncRNAs), including nuclear-enriched autosomal transcript 1 (NEAT1) and metastasis-associated lung adenocarcinoma transcript 1 (MALAT1, also called NEAT 2) (Tollervey et al., 2011). Expression of both lncRNAs is elevated in FTD patients with TDP-43 inclusions, thus correlating with their increased association with TDP-43 (Tollervey et al., 2011).

The binding of TDP-43 to small (<200 base) ncRNAs and miRNAs remains largely unexplored. However, the association of TDP-43 with Drosha microprocessor (Ling et al., 2010) and Dicer complexes (Freibaum et al., 2011; Kawahara and Mieda-Sato, 2012) provides a suggestive role of TDP-43 involvement in miRNA biogenesis. Indeed, let-7b miRNA is downregulated, whereas miR-663 is upregulated upon reduction of TDP-43 (Buratti et al., 2010). Di Carlo and collegues demonstrated that TDP-43 directly interacts with Drosha and controls its stability at different levels. Moreover, TDP-43 is also involved in the Drosha substrate recognition as in the regulation mediated by Drosha of Neurogenin 2, an important and master gene in neurogenesis (Di Carlo et al., 2013).

Fan et al. (2014) have performed CLIP-seq analysis to examine the small RNAs (pri-miRNAs, miRNAs, and piRNAs) bound to TDP-43 and found that a novel miRNA (miR-NID1), which is processed from the intron five of human neurexin 1 gene (*NRXN1*), interacts with TDP-43 and represses expression of *NRXN1*. Neurexins are cellular proteins that function as cell adhesion molecules and receptors in the vertebrate nervous system, involved in synaptic development including calcium signaling, heterogeneous cell-to-cell adhesion and synaptogenesis (Craig et al., 2006; Bottos et al., 2011) Disruptions or mutations of *NRXN1* have been reported to associate with autistic spectrum disorder (ASD), mental retardation, and schizophrenia (Reichelt et al., 2012).

Recent studies by King and colleagues identified a physical interaction between TDP-43 and miR-1 family which is known to be involved in smooth muscle gene repression in heart and an opposing myogenic differentiation (King et al., 2014). TDP-43 overexpression in skeletal muscle led to decrease of miR-1 and increased protein levels of the miR-1 family targets, IGF-1 and HDAC4. These results demonstrate that TDP-43 could influence miRNA regulation through a physical interaction by limiting their bioavailability for RISC loading and offer a mechanism by which mature miRNAs can be differentially regulated.

The expression of TDP-43 is tightly autoregulated through a complex interplay between transcription, splicing, and 3′ end processing (Avendaño-Vázquez et al., 2012): TDP-43 over-expression in humans and mice leads to activation of a 3′ UTR intron which results in excision of proximal polyA site (PAS) which in turn activates a cryptic PAS and prevents TDP-43 expression through a nuclear retention mechanism.

The above mentioned studies have highlighted that TDP-43 is linked to various mRNAs and non-coding RNAs, in a neuronal context wherein it mediates effects through splicing or interaction with Drosha and Dicer complexes. It is also involved in its autoregulation mediated at the RNA level.

Additionally, TDP 43 is known to interact with MATR3, a DNA RNA binding protein. Their interaction was confirmed to be RNA based. Mutations in this gene have been linked to cases of ALS. The authors further report that the phenotype observed in patients with MATR3 was a combination of those observed in cases of ALS and myopathy. Clinical symptoms were similar to patients with VCP mutations (Johnson et al., 2014).

## FUS

FUS, (fused in sarcoma, also called TLS: translocated in liposarcoma) belongs to the TET family of RNA binding proteins involved in many different cellular processes (Bertolotti et al., 1996; Law et al., 2006; Tan and Manley, 2009). *FUS*, located on chromosome 16 at locus p11.2, encodes a multifunctional protein able to bind and interact with single stranded RNA and double stranded DNA, participating in different aspects of RNA metabolism (Shelkovnikova et al., 2014).

### Structure

**Figure 3:**
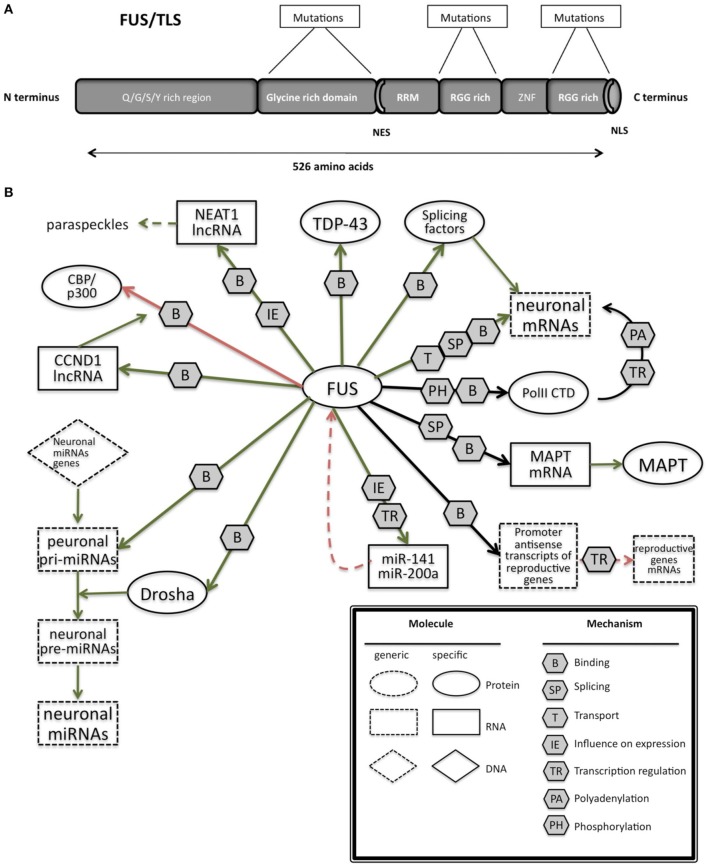
**(A)** Schematic representation of the functional domains in FUS/TLS. FUS contains a N-terminal part enriched in glutamine, glycine, serine and tyrosine residues (QGSY region), a glycine-rich region, a nuclear export signal (NES), an RNA recognition motif (RRM), repeats of arginine, glycine, glycine (RGG), a zinc finger motif (ZNF), and a C-terminal nuclear localization signal (NLS). Most of the mutation are localized in the glycine rich region and in the last 17 amino acids of the NLS part. **(B)** The network of interactions of FUS with proteins and RNAs. Green arrows indicate binding interactions or processes that result in activation or increased expression. Red arrows indicate binding interactions or processes that result in inhibition of activity or reduced expression. Black arrows indicate binding interactions or processes whose result can be either positive or negative. Dashed arrows indicated indirect processes. Symbols as in Legend. lncRNAs, long non-coding RNAs; IGF-1, insulin-like growth factor 1; HDAC4, histone deacetylase 4; NRXN1, neurexin 1; TDP-43, TAR DNA binding protein; FUS, fused in sarcoma; MAPT, microtubule-associated protein tau; NEAT1, nuclear-enriched autosomal transcript 1; CCND1, G1/S-specific cyclin-D1; CBP, CREB-binding protein; p300, Histone acetyltransferase p300; PolII CTD, Carboxy-terminal Domain of the RNA polymerase II.

FUS is characterized by different domains (Figure 3A): a N-terminal domain with transcriptional activating properties mainly composed of glutamine, glycine, serine, and tyrosine residues (Law et al., 2006), a glycine rich region, a RNA binding domain, and a highly conserved C-terminus capable of binding DNA, RNA and splicing factors (Law et al., 2006).

### Localization and function

FUS is mainly localized in the nucleus (Colombrita et al., 2009; Van Blitterswijk and Landers, 2010; Kawahara and Mieda-Sato, 2012) but it is also actively implicated in other cellular processes that occur in the cytoplasm such as mRNA transport, mRNA stability and translation (Buratti and Baralle, 2010; Colombrita et al., 2011). Indeed FUS was reported to shuttle between the nucleus and the cytoplasm, exporting to the cytoplasm spliced mRNAs in ribonucleoprotein complexes (Zinszner et al., 1997). Particularly, upon stimulation in hippocampal neurons FUS was reported to accumulate in the spines of mature dendrites, where local translation occurred (Fujii and Takumi, 2005). FUS immunoreactivity was also observed in dendritic spines in mature primary cultures and in adult hippocampus *in situ* (Belly et al., 2005; Table 1).

The C-terminal part of FUS encodes for a non-classic nuclear localization signal (Figure 3A; Iko et al., 2004) that is necessary for nuclear import, as it was demonstrated through the generation of deletion mutant lacking 13 amino acids in the C-terminal part of FUS(Dormann et al., 2010).

Several papers reported that mutations and aberrations of *FUS* are linked to the pathogenesis of frontotemporal degeneration (FTD) as well as familial and sporadic ALS (Kwiatkowski et al., 2009; Vance et al., 2009), as reported in Table 3. Moreover, FUS accumulates in inclusions in the cytoplasm of autopsied spinal cords and brains of sporadic and familial ALS and FTD. FUS inclusions are not only observed in presence of *FUS* mutations, as they were found in patients with different or unknown genetic defects such as sporadic ALS, ALS/dementia or FTLD (with or without progranulin mutations), FUS or TDP43 mutation-linked familial ALS, SOD1-negative familial ALS. These inclusions were also positive for TDP43/ubiquitin and p62 (Deng et al., 2010).

**Table 3:** **List of mutations in *FUS* and their characteristic phenotypes**.

**Subtypes of Dementia**	**Mutation**	**Change in amino acid**	**Type**	**References**
FTD	g.4961A>G	M254V	Unclear	Van Langenhove et al., 2010
bvFTD	g.31183985C>T	P106L	Unclear	Huey et al., 2012
ALS/FTD	g.31185031G>A	G206S	Unclear	Yan et al., 2010
FALS/PD/DE	g.31191418C>T	R521C	Unclear	Yan et al., 2010
ALS/FTD	g.31191419G>A	R521H	Pathogenic	Broustal et al., 2010

*ALS, Amyotrophic lateral sclerosis; FTD, Frontotemporal Dementia; bv FTD, behavioral variant Frontotemporal Dementia; FALS, Familial Amyotrophic lateral sclerosis; PD, Parkinson's disease; DE, Dementia*.

*All the information reported in the table is derived from a cumulative study of the literature and the database: http://www.molgen.ua.ac.be/ADMutations/default.cfm?MT=0&ML=2&Page=FTD*.

ALS/FTD patients show mutations mainly in the Glycine rich region and C-terminal part (Lagier-Tourenne et al., 2010). The mechanism underlying the pathogenesis of FUS mutations was related to FUS nucleus/cytoplasmic imbalance since ALS mutations increase its localization in the cytoplasm, observed through immunostaining of FUS in postmortem ALS brain samples (Kwiatkowski et al., 2009), or through the analysis in neuroblastoma cell lines of the subcellular localization of recombinant mutant FUS fused either to green fluorescent protein (GFP) (Kwiatkowski et al., 2009; Morlando et al., 2012), an N-terminal hemagglutinin (HA) tag (Vance et al., 2009), a C-terminal V5-His tag, or an N-terminal myc tag in HeLa (Ito et al., 2011).

Both the loss of FUS nuclear function and the potential gain of toxic effect by FUS in the cytoplasm could explain pathogenesis (Shelkovnikova et al., 2014).

Very few studies so far reported FTD cases associated with *FUS* mutations. The first analysis of *FUS* in FTD patients showed a novel missense mutation in the glycine-rich region of FUS, predicted to be pathogenic by *in silico* analysis (Van Langenhove et al., 2010). Subsequently another study found novel missense mutations in patients with familial ALS with features of frontotemporal dementia (FALS/FTD) and one with familial ALS with parkinsonism and dementia (FALS/PD/DE) (Yan et al., 2010). Recently, another study found two novel heterozygous missense mutations in *FUS* in patients with behavioral variant FTD (bvFTD), however the pathogenicity of these mutations needs to be further investigated in other screening (Huey et al., 2012).

FUS has been reported to co-localize with TDP-43 in nuclear complexes (Kim et al., 2010b; Ling et al., 2010) and in larger cytoplasmic complexes (Kim et al., 2010b). Purified FUS has also been reported to interact with purified His-tagged TDP-43 *in vitro* in an RNA-independent manner, associated to the C-terminal region of TDP-43 (Kim et al., 2010b). These ubiquitously expressed binding proteins seem to have similar and complementary functions.

Only the mutant form of FUS was found in stress granules in reponse to translational arrest (Bosco et al., 2010). FUS and TDP-43 were observed to co-localize in cytoplasmic aggregations of ALS/FTLD-affected neurons (Da Cruz and Cleveland, 2011). Dormann and colleagues found stress granule markers such as PABP-1 and eIF4G co-deposited with FUS inclusions in sections of post-mortem brain and spinal cord tissue from familial ALS-FUS and sporadic FTDLD-U. On the contrary, TDP inclusions did not show any co-localization with stress granules proteins in HeLa transiently transfected with the mutated form of FUS, after heat shock for 1 h (Dormann et al., 2010). Another study reported that ubiquitin-positive inclusions in frozen post-mortem tissue from FTLD-TDP patients were not stained with anti-FUS antibodies (Neumann et al., 2009b), therefore FUS and TDP-43 are not always found in the same inclusions or aggregates.

The relation between FUS and TDP-43 is reported as a delicate equilibrium, where small alteration on their relative quantity and presence in nucleus/cytoplasm could very likely cause serious problem over a long period (Colombrita et al., 2012), which might be an accumulation of events due to an alteration of their targetome.

### Implications of RNA in pathogenesis

FUS is involved in pre-mRNA splicing (Figure 3B), by interacting with splicing factors such as SRm160, PTB, and serine/arginine rich proteins (SR proteins) (Yang et al., 1998; Meissner et al., 2003). In addition the recent sequencing approaches applied to clarify the function and identify the targets of FUS reinforced its fundamental role in splicing (Colombrita et al., 2012) by revealing its binding to intronic sequences or to splice site acceptors.

Similarly to many other splicing factors, FUS can bind the C-terminal domain of RNA polymerase II and prevent the premature hyperphosphorylation of Ser2 in the C-terminal domain of RNA polymerase II. Moreover the lack of FUS leads to an accumulation of RNA polymerase II at the transcription start site with a shift toward abundance of mRNA isoforms with early polyadenylation (Schwartz et al., 2012).

FUS can bind to the promoter antisense strand transcript of some genes such as Ptprn2, Xrn1, Gak, or Glt1d1 and this interaction downregulates the transcription of the coding sense strand, but this effect seems to be specific for some genes enriched with GO terms connected to the reproductive process(Ishigaki et al., 2012).

As FUS was shown to regulate RNA polymerase II at many more gene promoters than the genes reported for splicing defects, its role on transcription could be a separated function in addition to the regulation on splicing (Schwartz et al., 2012). However, a small proportion of FUS target regions is localized in exonic sequences and in the 3′UTRs (Hoell et al., 2011), suggesting another potential role, such as the transport of mRNAs or the control of mRNA stability and translation (Fujii et al., 2005; Fujii and Takumi, 2005). A model was suggested, in which FUS is released from actin filaments, when cytoskeletal organization collapses, becoming free to be linked to the mRNA that is transported to the local translational machinery in the spines (Fujii and Takumi, 2005).

Recent techniques, like HITS-CLIP or RIP-CHIP were also used to identify FUS binding motif, but all the studies lead to the common assumption that FUS binds to specific secondary structures on its RNA targets and a primary sequences analysis is not sufficient (Colombrita et al., 2012; Ishigaki et al., 2012).

Interestingly, silencing of *FUS* was reported to alter splicing events in genes, such as *MAPT*, that have an important neuronal function (Ishigaki et al., 2012). This finding leads an unexpected connection between these two genes, both involved in the pathogenesis of FTD. In particular, FUS was shown to help the skipping of *MAPT* exon 10 in primary cortical neurons (Ishigaki et al., 2012). The alternative splicing of *MAPT* exon 10 is known to have a causative role in FTD as discussed later (*MAPT* paragraph).

FUS is also involved in microRNA biogenesis (Morlando et al., 2012), specifically interacting with pri-miRNAs and Drosha, and helping the recruitment of Drosha for the correct miRNA processing in neuronal cells. Several miRNAs like miR-9, miR-132, and miR-125b whose biogenesis is controlled by FUSare important for neuronal functions, neuronal differentiation, and synaptogenesis (Morlando et al., 2012). Additionally miR-9 and miR-132 have also been shown to control neurite extension and branching through downregulation of Foxp2 (Forkhead box protein P2) (Clovis et al., 2012) Moreover this role of FUS seems to be prominent in neuronal cells compared to non-neuronal cells, such as HeLa cells, in which the proportion of miRNAs affected by *FUS* knockdown was lower. Indeed the mutations known to induce a cytoplasmic delocalization of FUS would impede its nuclear role as pri-miRNA processor. Though the balance of nuclear and cytoplasmic FUS seems necessary, the sole role of nuclear FUS should not be neglected and further investigations would be needed to clarify its biological function within this cell compartment. Recently, the same laboratory demonstrated the presence of a regulatory loop in which FUS can enhance the expression of miR-141 and miR-200a, which in turn regulate FUS, through a binding on its 3′UTR. This pathway seems to be affected in the presence of one mutation found in two ALS patients (Dini Modigliani et al., 2014).

FUS is also reported to bind lncRNAs. The binding to lncRNA CCND1 induces an allosteric change in FUS, thus in turn permits its interaction with CBP/p300. As FUS represses CBP/p300-mediated transcription by inhibiting their histone acetyltransferase (HAT) functions (Wang et al., 2008a), in the presence of ncRNA CCDN1, CBP/p300-mediated transcription is repressed.

The nuclear-enriched abundant transcript 1 (NEAT1) produces two types of lncRNAs from the same promoter NEAT1_1 and NEAT1_2 (Nishimoto et al., 2013). FUS was shown to bind NEAT1_2, known to assemble and organize the core proteins of paraspeckles (Wang et al., 2008a; Hoell et al., 2011; Lagier-Tourenne et al., 2012), which represent a storage for the rapid release of RNAs during stress condition or a nuclear retention of long hyperedited transcripts (Prasanth et al., 2005; Chen and Carmichael, 2009). According to observations and data obtained from cultured cells, transgenic mice and human post-mortem tissue, paraspeckles represents an important protective cell mechanism during stress conditions (Nakagawa et al., 2011; Nakagawa and Hirose, 2012; Shelkovnikova et al., 2014).

Paraspeckels are present in almost all the cultured cells (Fox and Lamond, 2010), but in normal tissues are found only in cells that contain high levels of NEAT1_2 RNA and coherently, in neurons where NEAT1 is express at low levels, paraspeckles are not observed (Nakagawa et al., 2011).

The presence of FUS in paraspeckles was confirmed in different cell lines by three studies (Naganuma et al., 2012; Nishimoto et al., 2013; Shelkovnikova et al., 2014) Moreover, NEAT1 was shown through PAR-CLIP to be a target of both WT and mutant FUS (Hoell et al., 2011).

Paraspeckles are found in spinal motoneurons of patients at early stage of ALS. The possibility that aging induces an increase in the level of NEAT1_2 was ruled out due to the fact that human control cases were older that ALS cases of an average of 10 years. However, the process that induces an up-regulation of NEAT1_2 lncRNA during the early phases of ALS is still unknown (Nishimoto et al., 2013). Overall FUS seems to play a key role on the regulation of RNA at different levels, acting on transcription, splicing, transport, and stability of mRNA with a particular function in microRNA biogenesis and interaction with non-coding RNAs.

## *MAPT* (Tau)

*MAPT* (microtubule associated protein) encodes for protein Tau and is located on chromosome 17q21.3. The gene, which is 150 kb-long, contains 16 exons, out of which 11 are expressed in CNS (Wolfe, 2012).

### Structure

**Figure 4:**
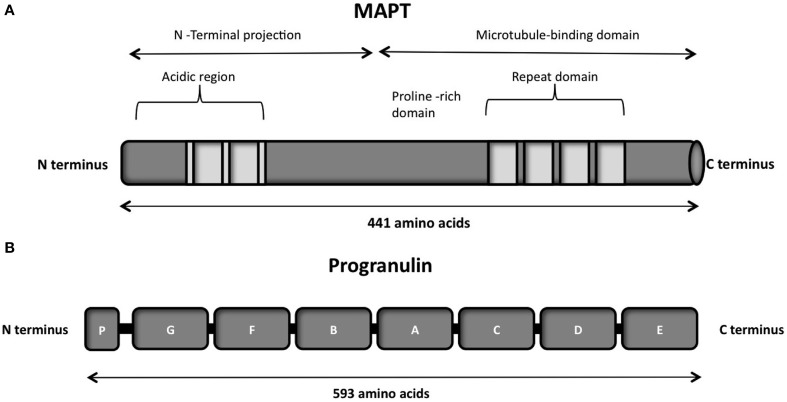
**(A)** Schematic representation of the functional domains of the largest tau isoform (441 amino acids 2N4R isoform). The N-terminal projection domain, including an acidic and a proline-rich region, interacts with cytoskeletal elements. The N-terminal part is also involved in signal transduction pathways by interacting with proteins such as PLC-γ and Src-kinases. The C-terminal part, also known as the microtubule-binding domain, regulates the rate of microtubules polymerization and is involved in binding with proteins such as protein phosphatase 2A (PP2A) or presenilin 1(PS1). **(B)** Schematic representation of the progranulin structure encoded by the major human transcript containing all the 13 exons. The lettered boxes on the progranulin scheme represent the individual granulin domains containing cystein-rich motifs.

The protein consists of a projection domain, including an acidic and a proline-rich region, which interacts with cytoskeletal elements (Figure 4A). The N-terminal part is involved in signal transduction pathways by interacting with proteins such as PLC-γ and Src-kinases. The C-terminal part, referred to as the microtubule binding domain, regulates the rate of microtubules polymerization and is involved in binding with functional proteins such as protein phosphatase 2A (PP2A) or presenilin 1 (PS1) (Luna-Muñoz et al., 2013).

### Localization and function

Tau is a microtubule-associated protein which is found in abundance in the axons of Central nervous system (CNS) and Peripheral nervous system (PNS) (Binder et al., 1985; Couchie et al., 1992; Table 1). It is also observed in astrocytes and oligodendrocytes in the CNS. The tau pre-mRNA undergoes alternative splicing at exons 2, 3, and 10 to give six different possible isoforms. Inclusion of exon 10 generates 4-repeat or 4R tau, while exclusion forms 3-repeat or 3R tau. In neurons this ratio is controlled throughout development, emphasizing the importance of this balance for neuronal functions.

### Implications of RNA in pathogenesis

In FTD populations, *MAPT* mutation frequency ranges from 8 to 50%. To date, 44 different *MAPT* mutations, either mis-sense or splice mutations or both, have been discovered in 138 different families (Cruts et al., 2012). The list of pathogenic mutations observed in *MAPT* are reported in Table 4). Most missense mutations alter ability of tau to bind to microtubules, thus leading to the formation of inclusion in neurons and glia, called neurofibrillary tangles (NFT) (Lee et al., 2005).

**Table 4:** **List of mutations in *MAPT* and their characteristic phenotypes**.

**Subtypes of dementia**	**Mutation**	**Change in amino acid**	**Type**	**References**
FTD	g.75756G>A	R5H	Pathogenic	Hayashi et al., 2002
FTD	g.110018A>C	K592T	Pathogenic	Rizzini et al., 2000
FTD	g.110026A>G	I595V	Pathogenic	Grover et al., 2003
FTD	g.110044C>G	L601V	Pathogenic	Kobayashi et al., 2003
FTD	g.110063G>T	G607V	Pathogenic	Schenk, 1959
FTD	g.110065G>A	G608R	Pathogenic	Van der Zee et al., 2006
FTD	g.123725T>G	N614K	Pathogenic	Wszolek et al., 1992
FTD	g.123729_123731delAAG	DeltaK616 (alias ΔK280; ΔK281)	Unclear	Rizzu et al., 1999
FTD	g.123740T>C	L619	Pathogenic	D'Souza et al., 1999
FTD	g.123774A>C	N631H	Pathogenic	Iseki et al., 2001
FTD	g.123776T>C	N631	Pathogenic	Brown et al., 1996
FTD	g.123789C>A	P636T	Pathogenic	Lladó et al., 2007
FTD	g.123789C>T	P636S	Pathogenic	Bugiani et al., 1999
FTD	g.123790C>T	P636L	Pathogenic	Hutton et al., 1998
FTD	g.123802G>A	S640N	Pathogenic	Iijima et al., 1999
FTD	g.123802G>T	S640I	Pathogenic	Kovacs et al., 2008
FTD	g.123803T>C	S640	Pathogenic	Spillantini and Goedert, 2000
FTD/PSP	g.123806G>A	Intronic	Pathogenic	Spillantini et al., 1997
FTD	g.123814T>C	Intronic	Pathogenic	Miyamoto et al., 2001
FTD	g.123815C>T	Intronic	Pathogenic	Takamatsu et al., 1998
FTD	g.123816A>G	Intronic	Pathogenic	Hutton et al., 1998
FTD	g.123817C>T	Intronic	Pathogenic	Lynch et al., 1994
FTD	g.123819C>T	Intronic	Pathogenic	Lanska et al., 1994
FTD	g.123822C>G	Intronic	Pathogenic	Stanford et al., 2003
FTD	g.127672T>G	L650R	Pathogenic	Rosso et al., 2003
FTD	g.127673G>A	L315	Pathogenic	(Bird, 2005, Personal Communication)
FTD	g.127678A>T	K652M	Pathogenic	Zarranz et al., 2005
FTD/PD/MND	g.127687C>T	S655F	Pathogenic	Rosso et al., 2002
FTD	g.132033G>A	G670S	Pathogenic	Spina et al., 2007
FTD	g.132034G>T	G670V	Pathogenic	Neumann et al., 2005
FTD	g.137420G>A	G389R	Pathogenic	Pickering-Brown et al., 2000
FTD	g.137420G>C	G389R	Pathogenic	Murrell et al., 1999
FTD	g.137471C>T	R741W	Pathogenic	Dickson, 1997
FTD	g.137525C>A	Q424K	Pathogenic	(Brice, 2005, Personal Communication)
FTD	g.137535C>T	T762M	Pathogenic	Giaccone et al., 2004
bvFTD	c.163G>A	G55R	Pathogenic	Iyer et al., 2013
FTD	c.363T>C	V363A	Pathogenic	Rossi et al., 2014
FTD	c.363C>A	V363I	Pathogenic	Rossi et al., 2014
FTD	c.454G>A	A152T	Pathogenic	Kara et al., 2012
FTD	c.530A>T	D177V	Unclear	Kim et al., 2014
FTD	c.853A > C	S285R	Pathogenic	Ogaki et al., 2013
FTD	c.892 A>G	K298E	Pathogenic	Iovino et al., 2014
FTD	c.1090C>T	P364S	Pathogenic	Rossi et al., 2012
FTD	c.1096G>A	G366R	Pathogenic	Rossi et al., 2012
FTD	c.1228A>C	N410H	Pathogenic	Kouri et al., 2014
FTD	c.1381-74G > A	Intronic	Pathogenic	Kim et al., 2010a
FTD	c.1908G > A	P636P	Pathogenic	Kim et al., 2010a
FTD	c.1815G > A	P605P	Pathogenic	Kim et al., 2010a
FTD	c.1828-47C > A	Intronic	Pathogenic	Kim et al., 2010a
FTD	c.2002+90G > A	Intronic	Pathogenic	Kim et al., 2010a
FTD	c.2092G>A	V363I	Pathogenic	Bessi et al., 2010
FTD	IVS10+4A>C	Intronic	Pathogenic	Anfossi et al., 2011
FTD	IVS9-15T>C	Intronic	Pathogenic	Anfossi et al., 2011
FTD	g.132037A>G	Q336R	Pathogenic	Pickering-Brown et al., 2004

*FTD, Frontotemporal Dementia; bv FTD, behavioral variant frontotemporal Dementia*.

*All the information reported in the table is derived from a cumulative study of the literature and the database: http://www.molgen.ua.ac.be/ADMutations/default.cfm?MT=0&ML=2&Page=FTD*.

About half of the mutations in *MAPT*, however, are associated with alteration of splicing of exon 10 and increase the ratio of 4R to 3R. The mutations near exon 10 5′splice site enhance inclusion of exon 10 either by altering the linear *cis*-splicing elements or by destabilizing a stem-loop structure at the exon-intron junction (D'Souza et al., 1999; Grover et al., 1999; Spillantini and Goedert, 2013). This stem-loop arises as a result of the self complementarity among bases in this region and has a putative role in masking the 5′ splice site Mutations that disrupt the stem-loop structure make the 5′ splice site accesible to splicing factors, leading to inclusion of exon 10 (Wolfe, 2012).

Though mutations lead to alteration of splicing at the mRNA level, their primary effect becomes pathogenic through changes in the protein level in about half of the cases (Goedert and Jakes, 2005).

The human *MAPT* 3′UTR, as well as that of rodents, contains two Polyadenylation Signals (PAS) in tandem and can undergo alternative polyadenylation (APA) to produce transcripts of approximately 2 or 6 kb, namely the short and long transcript variants (Poorkaj et al., 2001). Dickson and colleagues investigated the role of human *MAPT* 3′-UTR in regulating tau expression (Dickson et al., 2013). They observed that the two *MAPT* 3′UTR variants are differentially regulated and influence both mRNA stability and protein expression levels. The same authors have reported that miR-34a can bind the human *MAPT* 3′-UTR long form and reduce tau levels, whereas inhibition of endogenous miR-34 family members leads to increased tau levels, leading to a hypothesis that up-regulation of miR-34 observed during neuronal differentiation could be a compensatory mechanism to decrease the expression of tau aggregates. Recent work (Wu et al., 2013a) also confirms the finding that *MAPT* is regulated by miRNA 34c-5p and miRNA 34c-3p, which bind to its 3′UTR.

Additionally, work by Zovoilis and colleagues have suggested that miR-34c could be a marker for the onset of cognitive disturbances linked to Alzheimers disease and they also indicate that targeting miR-34c could thus be a suitable therapy (Zovoilis et al., 2011).

Studies also reported that miR-34 regulates apoptosis by blocking the SIRT1 gene (Hermeking, 2010) and astrocytic apoptosis has been observed as an early event in FTLD conditions (Broe et al., 2004). These findings suggest that miRNAs might be involved in FTD through apoptotic mechanisms.

Tau is known to spread through synaptic and non-synaptic mechanisms (Medina and Avila, 2014) and its accumulation is thought to be mediated through spreading of the protein from cell to cell. Tau has been reported to be secreted unconventionally in naked form (Chai et al., 2012) or associated to exosomes (Saman et al., 2012) and/or other membrane vesicles (Simón et al., 2012). This method of elimination of tau has been suggested as a response mechanism to inhibit tau secretion and toxicity. Recent reports have suggested that tau is released into culture medium from neuroblastoma cells, tau-expressing non-neuronal cells, induced pluripotent stem cell-derived human neurons, and mouse primary neurons (Kim et al., 2010a; Shi et al., 2012). This has also been observed in the brain interstitial fluid of both wild-type and P301S tau-expressing mice in micro-dialysis studies (Yamada et al., 2011). Clinico-pathological studies underline the tau pathology progression from entorhinal cortex through the hippocampus and into limbic system (Arriagada et al., 1992). Recent *in vivo* studies in tauopathy transgenic mouse models have also highlighted the spreading of tau pathology through a trans-synaptic mechanism in anatomically connected neuronal networks (De Calignon et al., 2012; Liu et al., 2012). Apart from these, intracerebral inoculation of synthetic tau fibrils induced NFT (Neuro fibrillary tangles) like inclusions that propagated from injected sites to other connected regions of brain (Iba et al., 2013).

Current hypotheses also include that pathological progression of improperly folded of tau could be transferred between neuronal cells via a prion-like seeding mechanism which might lead to neurodegeneration.

The major implication observed upon mutations which lead to splice defects highlights the relevance of regulation at RNA level which decides the fate of onset of neurodegeneration. The regulation of MAPT mediated through miRNAs further indicates the role of non-coding RNAs in determining tau protein levels.

## *GRN* (progranulin)

*GRN* is located on the long arm of chromosome 17 at the locus q21.31 which is present at a distance of 1.7 Mb from *MAPT* (Baker et al., 2006; Cruts et al., 2006). *GRN* encodes for a 593 aa precursor protein of 68.5 kDa called progranulin.

### Structure

Progranulin can be N-glycosylated at five potential sites and secreted as a mature protein of 88 kDa (Chen-Plotkin et al., 2010; Songsrirote et al., 2010). The protein is formed by 7.5 cysteine-rich granulin domains, separated through linker sequences that contain disulfide bridges (He and Bateman, 2003), as represented in Figure 4B. This characteristic structure can be cleaved at the intra-linker spacer sequences to produce seven non-identical granulins that contain cysteine-rich motifs. Different proteases can cleave progranulin, such as matrix metalloproteinase-14 (Butler et al., 2008), elastase (Zhu et al., 2002), proteinase 3, and neutrophil elastase (NE) at the pericellular microenvironment of the neutrophil cell surface (Kessenbrock et al., 2008). The full-length progranulin, once secreted, is protected from cleavage by the high-density lipoprotein (HDL)/Apolipoprotein A-I complex (Okura et al., 2010) and the secretory leukocyte protease inhibitor (SLPI) (Zhu et al., 2002).

### Localization and function

Progranulin is present in many tissues, is highly expressed in immune system cells (Daniel et al., 2000) and in a medium level in the brain (Bhandari et al., 1996; Ahmed et al., 2007), where it is highly expressed in specific populations of neuronal cells, such as cortical neurons, and granule cells of the hippocampus (Daniel et al., 2000; Table 1). The subcellular location of progranulin seems to be the endoplasmic reticulum (ER) and Golgi, where it is particular abundant in mouse cortical neurons and mouse microglia (Almeida et al., 2011). Progranulin is implicated in a wide range of biological processes such as embryogenesis (Díaz-Cueto et al., 2000; Daniel et al., 2003; Bateman and Bennett, 2009), cell survival and cell growth (Plowman et al., 1992; He and Bateman, 1999), inflammation and wound repair (Zhu et al., 2002; He et al., 2003; Kessenbrock et al., 2008; Yin et al., 2010), transcriptional repression (Hoque et al., 2003, 2010) and several reports suggest its role in neuronal development (Van Damme et al., 2008). Interestingly, progranulin and the proteolytically cleaved granulins can have coherent functions, such as in the regulation of neurite outgrowth (Van Damme et al., 2008), or they can have contrasting roles, such as in inflammation processes (He and Bateman, 2003).

To date, 69 different *GRN* mutations have been discovered in 231 families (Cruts et al., 2012). A list of detailed pathogenic mutations are reported in Table 5. The *GRN* mutations frequency range from 1 to 11.7% in FTD patients, but the frequency rises to 12–25% in familial FTD (Cruts et al., 2006; Gass et al., 2006; Huey et al., 2006; Bronner et al., 2007; Borroni et al., 2008). There are different types of *GRN* mutations, the majority are classified as non-sense, frameshift, and splice site mutations that cause a premature stop codons (Baker et al., 2006; Cruts et al., 2006). However, the pathogenic variants include also missense mutations with a partial decrease of progranulin and a loss of its function (Mukherjee et al., 2006, 2008; Shankaran et al., 2008; Wang et al., 2010). Silent and intronic mutation with unknown pathology can also occur. Generally the pathogenic *GRN* mutations lead to a decreased *GRN* expression due to a non-sense-mediated mRNA decay, resulting in a *GRN* haploinsufficiency inherited in an autosomal dominant manner (Baker et al., 2006; Cruts et al., 2006; Gass et al., 2006; Cruts and Van Broeckhoven, 2008).

**Table 5:** **List of mutations in *GRN* and their characteristic phenotypes**.

**Subtypes of Dementia**	**Mutation**	**Change in amino acid**	**Type**	**References**
FTD	delGRN[DR184]	Complete gene deletion	Pathogenic	Gijselinck et al., 2008
FTD	c.-7-20C>T	INTRON	Suggesting Pathogenic	Kim et al., 2010a
FTD	c.349 + 34C > T	INTRON	Suggesting Pathogenic	Kim et al., 2010a
FTD	IVS6+5_8delGTGA	N/A	Unclear	Marcon et al., 2011; Skoglund et al., 2011
FTD	c.1138C>G	Q380E	Unclear	Kim et al., 2014
FTD	g.2988_2989delCA	P439_R440fsX6	Pathogenic	Gabryelewicz et al., 2010
FTD	g.5215A>T	Complete protein degradation	Pathogenic	Le Ber et al., 2007
FTD	g.5217G>C	Complete protein degradation	Pathogenic	Cruts et al., 2006
FTD	g.5913A>G	INTRON	Pathogenic	Mukherjee et al., 2008
FTD/PD	g.8948_12532del	Complete protein deletion	Pathogenic	Rovelet-Lecrux et al., 2008
FTD	g.9044T>C	Predicted failed translation	Pathogenic	Baker et al., 2006
FTD	g.9045G>A	Predicted failed translation	Pathogenic	Cruts et al., 2006
FTD/MND	g.9055G>C	V5L	Unclear	Lopez de Munain et al., 2008
FTD	g.9061T>C	W7R	Unclear	Le Ber et al., 2007
FTD/PPA	g.9068C>A	A9D	Pathogenic	Mukherjee et al., 2006
FTD	g.9132_9133insCTGC	C31LfsX35	Pathogenic	Baker et al., 2006
FTD/PPA	g.9144delC	G35EfsX19	Pathogenic	Gass et al., 2006
FTD	g.9181G>A	Failed translation initiation	Pathogenic	Gass et al., 2006
FTD/AD	g.9319delA	T52HfsX2	Pathogenic	Gass et al., 2006
FTD	g.9399_9400delAG	G79DfsX39	Pathogenic	Gass et al., 2006
FTD	g.9408delC	S82VfsX174	Pathogenic	Bronner et al., 2007
FTD	g.9429G>A	E88	Unclear	Gass et al., 2006
FTD	g.9593T>C	C105R	Unclear	Gass et al., 2006
FTD	g.10129C>T	Q125X	Pathogenic	Baker et al., 2006
FTD	g.10134 C>G	C126W	Unclear	Bernardi et al., 2012
FTD	g.10136_10137delCT	P127RfsX2	Pathogenic	Cruts et al., 2006
FTD	g.10144_10147delCAGT	Q130SfsX125	Pathogenic	Baker et al., 2006
FTD	g.10319G>A	A155WfsX56	Pathogenic	Gass et al., 2006
FTD/PPA	g.10645_10646delCA	S226WfsX28	Pathogenic	Gass et al., 2006
FTD	g.10668C>A	P233Q	Unclear	Bronner et al., 2007
FTD	g.10678C>T	N236	Unclear	Gass et al., 2006
FTD	g.10679G>C	V200GfsX18	Pathogenic	Gass et al., 2006
FTD	g.10965_10966delTG	C253X	Pathogenic	Gass et al., 2006
FTD	g.11002 G>C	A266P	Unclear	Bernardi et al., 2012
FTD	g.11041_11042insCTGA	A237WfsX4	Pathogenic	Cruts et al., 2006
FTD/CBS	g.11240G>C	V279GfsX5	Pathogenic	Gass et al., 2006
FTD	g.11266G>C	E287D	Unclear	Gass et al., 2006
FTD	g.11315_11316insTG	W304LfsX58	Pathogenic	Gass et al., 2006
FTD	g.11316G>A	W304X	Pathogenic	Gass et al., 2006
FTD	g.11339G>A	V279GfsX5	Pathogenic	Baker et al., 2006
FTD/CBS	g.11639delC	T382SfsX30	Pathogenic	Baker et al., 2006
FTD	g.11651G>A	W386X	Pathogenic	Baker et al., 2006
FTD	g.11944_11945delGT	V411SfsX2	Pathogenic	Bronner et al., 2007
FTD	g.11965C>T	R418X	Pathogenic	Baker et al., 2006
FTD	g.12054C>T	H447	Unclear	Bronner et al., 2007
FTD	g.12108_12109insC	C466LfsX46	Pathogenic	Gass et al., 2006
FTD	g.12115C>T	Q468X	Pathogenic	Baker et al., 2006
FTD	g.12227C>T	C474	Unclear	Gass et al., 2006
FTD	g.12282C>T	R493X	Pathogenic	Huey et al., 2006
FTD	g.12428G>C	W541C	Unclear	Bronner et al., 2007

*AD, Alzheimers disease; CBS, Corticobasal syndrome; FTD, Frontotemporal Dementia; PPA, Primary progressive aphasia; MND, Motor neuron disease; PD, Parkinsons disease*.

*All the information reported in the table is derived from a cumulative study of the literature and the database: http://www.molgen.ua.ac.be/ADMutations/default.cfm?MT=0&ML=2&Page=FTD*.

Indeed progranulin levels, measured in either the serum or cerebrospinal fluid (CSF) of patients with *GRN* mutations are ~30–50% of normal (Van Damme et al., 2008). Moreover, a decreased progranulin level can be also detected in plasma of *GRN* mutations patients (Finch et al., 2009) and a reduced GRN mRNA level can be observed in patient whole blood samples through microarray experiments (Coppola et al., 2008). In contrast an increased level of GRN mRNA was observed in the frontal cortex from post-mortem brain samples of FTD patients with *GRN* mutations, as compared to FTD patients without *GRN* mutations (Chen-Plotkin et al., 2010). The higher level of GRN transcripts could be due to the robust microglia infiltrations, observed in the brain tissues of *GRN* mutation patients. Indeed microglia shows high level of GRN expression.

### Implications of RNA in pathogenesis

Most of the patients with FTLD-U show *GRN* mutations with presence of TDP-43 ubiquitin positive inclusions, hence bearing the term FTLD-TDP (Mackenzie et al., 2006, 2010; Sampathu et al., 2006). The relation between TDP-43 and progranulin is not fully understood, however several recent studies indicate that TDP-43 controls the expression of progranulin by binding to GRN mRNA. On a study in which TDP-43 targets were identified through a RIP-chip analysis, it is shown that TDP-43 has a post-transcriptional regulation on GRN and VEGFA (Vascular endothelial growth factor A) (Colombrita et al., 2012).

As previously mentioned, TDP-43 was shown to specifically bind GRN 3′UTR controlling GRN mRNA stability and the final quantity of progranulin protein (Polymenidou et al., 2011; Colombrita et al., 2012). Moreover a knock-down of TDP-43 in mice showed an increase in the amount of GRN mRNA level (Polymenidou et al., 2011; Colombrita et al., 2012). Depletion of TDP-43 also led to altered splicing of sortilin, the putative progranulin receptor (Polymenidou et al., 2011). The relation between GRN and TDP-43 was also demonstrated *in vitro*: cells that were treated with siRNA against GRN for 72 h, showed a caspase-dependent cleavage of TDP-43 into fragments (Zhang et al., 2007); whereas primary neuronal cultures upon knowckdown of GRN showed a re-localization of TDP-43 in the cytoplasm (Guo et al., 2010).

Through genetic association analysis, a common genetic variation localized on the 3′UTR of GRN (rs5848) was shown to represent a genetic risk factor for FTD (Rademakers et al., 2008). Progranulin levels in brain extracts from rs5848 TT homozygous FTD patients were lower than in CC carriers, as observed through western blot analyses, ELISA, and immunohistochemistry. A stronger binding of miR-659 in the 3′UTR of GRN was shown in the presence of the rs5848 T variant, and might explain the reduced progranulin levels.

It is reported that miR-107 is downregulated in presence of Alzheimer's disease at early stage (Wang et al., 2008b). Another study demonstrated through a RIP-Chip analysis performed in human H4 neuroglioma cells that the open reading frame of GRN mRNA contains many recognizing sequences elements for miR-107 (Wang et al., 2010), showing implications of miR-107 deregulation in neurodegenerative diseases. In particular miR-107 regulation of GRN seems to be relevant to glucose metabolism in cultured H4 neuroglioma cells. Previous analysis identified miR-107 as one of the microRNAs that increase their expression with glucose supplementation in cell culture medium (Tang et al., 2009). Wang and colleagues reported that glucose metabolic pathway may recruit miR-107 to regulate GRN expression. Another microRNAs that was found significantly down-regulated in brains of Alzheimer's disease patients is miR-29b, that beloged to the miR-29a/b-1 cluster (Hébert et al., 2008). Interestingly progranulin can also be regulated by miR-29b through a binding in the 3′UTR of GRN mRNA (Jiao et al., 2010). It would be useful to know if these microRNAs deregulation can contribute to the pathogenesis of dementia. So far different microRNAs seem to be important for the control of progranulin along with the role played by TDP-43 on the stability of GRN mRNA and its expression.

## VCP

The VCP (Valosin-containging protein) gene is located on chromosome 9p13.3. It also called p97 or CDC48, consists of 17 coding exons.

### Structure

**Figure 5:**
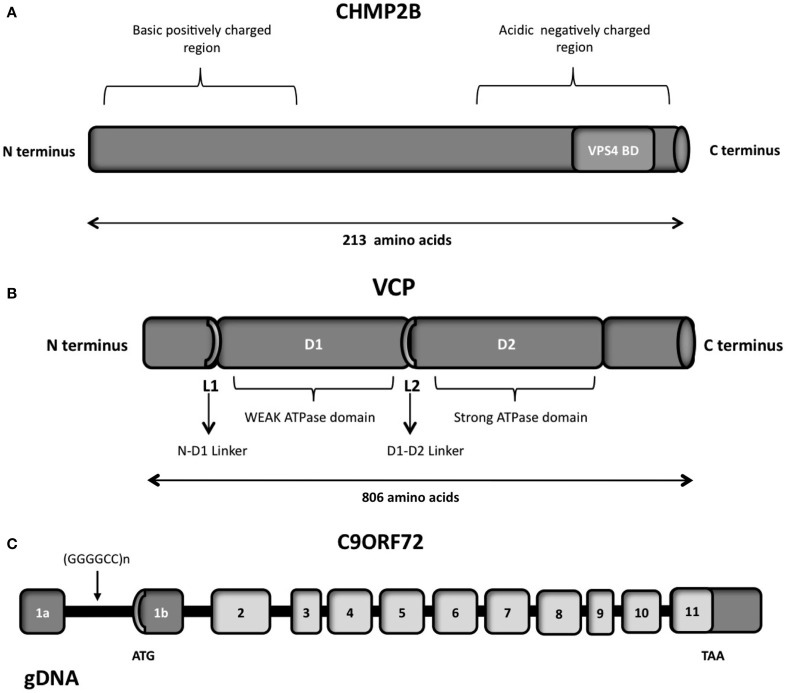
**(A)** Schematic representation of the CHMP2B which contains an acidic negatively charged C-terminal domain and a basic positively charged N-terminal domain, a predicted coiled-coil structure (14-51 aa) and a conserved Snf7 domain (16-178 aa). The autoinhibitory structure formed in the cytosol due to the C- and N- terminal part interactions is reverted through the binding of VSP4 on the VPS4 binding domain (VPS4 BD), localized on the C-terminal part. **(B)** Schematic representation of the six functional domains of the VCP protein: the N-terminal domain, the weak ATPase domain (D1), the major ATPase domain (D2), the N-D1 linker domain, the flexible D1–D2 linker domain and the C-terminal domain. **(C)** Overview of the genomic structure of the *C9ORF72* gene, with white boxes representing the coding exons and gray boxes representing the non-coding exons. The position of the hexanucleotide repeat (GGGGCC), the start codon (ATG), and the stop codon (TAA) are indicated in the scheme.

The VCP protein is composed of four domains vital for its proper functioning, namely the N, D1 D2 and C-terminal domains (Figure 5B; DeLaBarre et al., 2006; Pye et al., 2007). The VCP N domain is encoded by exons 1, 2, 3, 4 and 5, while the D1 and D2 domains are encoded by exons 6, 7, 8, 9, 10 and 12, 13, 14, respectively. There are two linker domains in the protein: the N-D1 linker and the flexible D1-D2 linker.

VCP is a member of the AAA-ATPase gene superfamily (ATPase Associated with diverse cellular Activities) (Woodman, 2003; Wang et al., 2004b), and is one of the most abundant cytosolic proteins (Table 1) conserved throughout in mammals. The complete protein contains 806 amino acids. The N domain of VCP is responsible for the cofactor and ubiquitin binding function (Wang et al., 2004b). While the D1 domain mediates oligomerization-independent nucleotide binding, the D2 domain confers most of the ATPase activity (Wang et al., 2004b).

### Localization and function

This protein functions as a molecular chaperone in various distinct cellular processes including ubiquitin-dependent protein degradation, stress responses, programmed cell death, nuclear envelope reconstruction, and Golgi and endoplasmic reticulum (ER) assembly (Guinto et al., 2007).

VCP is known to be involved in protein aggregation/quality control of mitochondria and cell proliferation (Hayashi, 2013) and is vital for retro-translocation of misfolded proteins from Endoplasmic reticulum to cytoplasm (Kimonis et al., 2008). Mutation and depletion studies of VCP have provided evidence of accumulation of poly-ubiquitinated proteins (Dai and Li, 2001). Mutations in this gene may suggest the disruption of normal protein degradation pathway in the disease. This could be facilitated through the disruption of binding between the VCP and protein adaptors.

The expression of mutant VCP in myoblastic cell lines is associated with increased ubiquitin conjugated proteins (Weihl et al., 2006). Studies on overexpression of mutant VCP protein in transgenic mice implicated an age-dependent muscle weakness and Ubiquitin-positive inclusions and accumulation of high molecular weight protein aggregates (Weihl et al., 2007).

VCP functions as a homohexamer (Zhang et al., 2000; Rouiller et al., 2002) by binding to multiple proteins associated with Ubiquitin proteasome system (UPS). The VCP complex binds to polyubiquitin chains and unbounds ubiquitinated proteins from their binding partners thereby facilitating transport to the UPS.

### Implications of RNA in pathogenesis

To date, 18 different VCP mutations have been discovered in 48 different families, which include FTLD that is associated with ALS, inclusion body myopathy, and Paget disease (Cruts et al., 2012). Table 6 high-lights the list of pathogenic mutations observed so far. The association of inclusion body myopathy and FTD was established by Kovach et al. (2001).

**Table 6:** **List of mutations in *VCP* and their characteristic phenotypes**.

**Subtypes of Dementia**	**Mutation**	**Change in amino acid**	**Type**	**References**
IBMPFD	g.284G>A	R92H	Unclear	Kaleem et al., 2007
IBMPFD	g.410C>T	P137L	Pathogenic	Stojkovic et al., 2009
IBMPFD	g.4438C>T	R93C	Pathogenic	Guyant-Maréchal et al., 2006
IBMPFD	g.4444C>G	R95G	Pathogenic	Watts et al., 2004
IBMPFD	g.4444C>T	R95C	Pathogenic	Kimonis et al., 2008
IBMPFD	g.6990C>T	R155C	Pathogenic	Watts et al., 2004
IBMPFD	g.6991G>A	R155H	Pathogenic	Watts et al., 2004
IBMPFD	g.6991G>T	R155L	Unclear	Kumar et al., 2010
IBMPFD	g.6991G>C	R155P	Pathogenic	Watts et al., 2004
IBMPFD	g.6990C>A	R155S	Pathogenic	Stojkovic et al., 2009; Vesa et al., 2009
IBMPFD	g.6996G>C	G157R	Pathogenic	Stojkovic et al., 2009; Djamshidian et al., 2009
IBMPFD	g.7002C>T	R159C	Pathogenic	Bersano et al., 2009
IBMPFD	g.7003G>A	R159H	Pathogenic	Haubenberger et al., 2005
IBMPFD	g.7099G>A	R191Q	Pathogenic	Watts et al., 2004
IBMPFD	g.8085T>G	L198W	Pathogenic	Watts et al., 2007
IBMPFD	g.8187C>A	A232E	Pathogenic	Watts et al., 2004
IBMPFD	g.9349A>G	T262A	Pathogenic	Spina et al., 2008
IBMPFD	g.10742A>C	N387H	Pathogenic	Watts et al., 2007
IBMPFD	g.11295G>C	A439P	Pathogenic	Shi et al., 2012
IBMPFD	g.11295G>T	A439S	Pathogenic	Stojkovic et al., 2009

*IBMPFD, Inclusion body myopathy with Paget's disease of the bone and frontotemporal dementia*.

*All the information reported in the table is derived from a cumulative study of the literature and the database: http://www.molgen.ua.ac.be/ADMutations/default.cfm?MT=0&ML=2&Page=FTD*.

A recent work by Jacquin et al. (2013) has showed R155H (464 G>A) mutation of the *VCP* gene in a French family, led to the Inclusion body myopathy with Paget's disease of the bone and frontotemporal dementia (IBMPFD), with a psychiatric onset of FTD.

The expression of IMBPFD-associated *VCP* mutations in skeletal muscle cells reduced UNC-45 (a molecular chaperone involved in myosin assembly) degradation that is linked to severe myofibril disorganization in myotubules. This study suggests a possible mechanism for the selective vulnerability of skeletal muscle in IBMPFD; however, the implication for the pathogenesis of FTD still remains unknown. Studies on a *VCP*-mutant transgenic mouse have shown TDP-43 and ubiquitin positive accumulations (Custer et al., 2010) suggesting a possible interplay between these proteins. IBMPFD is known to have TDP-43 aggregation with *VCP* mutations (Nalbandian et al., 2011). Ju et al. (2009) have established a link between *VCP* and autophagosomes wherein the loss of VCP leads to accumulation of autophagosomes, thus establishing a possible cause of aggregation of proteins such as TDP-43.

VCP has been detected in a few inclusions of neurodegenerative diseases such as senile plaques in Alzheimer's disease, Lewy bodies in Parkinson's disease, neuronal intranuclear inclusions in CAG/polyglutamine diseases and ubiquitin-positive inclusions in ALS (Hirabayashi et al., 2001; Mizuno et al., 2003; Ishigaki et al., 2004).

Bartolome and colleagues have performed analyses in fibroblasts derived from patients with three different pathogenic *VCP* mutations, VCP-deficient cells, mouse cortical primary neurons and astrocytes, to conclude that reduction of VCP led to uncoupling of mitochondria and increased oxygen consumption and a subsequent decrease in ATP of cells leading to cellular toxicity and neuronal death (Bartolome et al., 2013).

VCP has been recently involved in clearance of mRNP granules (Buchan et al., 2013), thereby unraveling a new mechanism in clearance of RNPs from the cell. This might indicate why *VCP* mutations lead to accumulation of stress granule constituents or cytoplasmic inclusions. mRNP granules assemble to form stress granules as a consequence of their aggregation (Erickson and Lykke-Andersen, 2011). Wang et al. (2015) have shown a direct interaction between VCP and FUS. VCP being a key regulator of protein degradation, DNA interaction, and mitochondrial activity, its direct interaction with FUS is intriguing. Although there is no evidence which shows a direct interaction or implication of *VCP* mutations on RNA, its association with TDP-43 and FUS, both of which are RNA binding proteins may suggest their unraveled interactions in RNA metabolism.

## CHMP2B

CHMP2B (Charged multivesicular body protein) is encoded by a gene located on chromosome 3 and is a component of the endosomal sorting complex required for transport-III (ESCRT-III complex).

### Structure

CHMP2B is a protein of 213 amino acids, with an acid C-terminal domain and basic N-terminal domain (Figure 5A), and a predicted coiled-coil structure (Skibinski et al., 2005). The negatively charged C-terminal domain interacts with the positively charged N-terminal part, creating a closed and autoinhibitory structure in the cytosol (Whitley et al., 2003; Shim et al., 2007). CHMP2B requires therefore an activation process performed by VPS4, which binds to its C-terminal domain. Indeed the C-terminal part of CHMP2B contains a binding domain for VPS4. VPS4 is a member of the AAA-ATPase family and it has a role in catalyzing the dissociation of ESCRT complexes from endosomes (Katzmann et al., 2002). The ATPase activity of VPS4 is important for endosomal sorting (Katzmann et al., 2002; Obita et al., 2007). Mutations localized in the VSP4-binding region impair the function of CHMP2B, preventing the formation of protrusions from the cell (Bodon et al., 2011).

### Localization and function

Northern-Blot analysis demonstrated that CHMP2B is expressed in all the major parts of the brain, including the temporal and frontal lobes (Table 1). Moreover through *in situ* hybridization of mouse brain, an enhanced expression of CHMP2B in the hippocampus, frontal and temporal lobes, and cerebellum was shown (Skibinski et al., 2005). The endosomal-sorting complex required for transport (ESCRT) is a protein complex involved in the endocytosed protein trafficking from endosome to lysosomes, playing an important role for sorting of proteins and for their efficient lysosomal degradation (Urwin et al., 2010). Moreover ESCRT complexes have a relevant role at the plasma membrane, during cytokinesis (Carlton and Martin-Serrano, 2007; Elia and Sougrat, 2011), budding of some enveloped viruses (Usami et al., 2009; Martin-Serrano and Neil, 2011), autophagy and transcriptional regulation (Roxrud et al., 2010; Schmidt and Teis, 2012). Endosomal trafficking is a key process for the internalization and transport of neuronal growth factors, secreted growth factors, signaling molecules (Bronfman et al., 2007). A dysfunction in this process could lead to an altered cell-signaling and aberrant communication between cells.

### Implications of RNA in pathogenesis

In particular ESCRT dysfunction is associated with neurodegeneration, indeed mutation in *CHMP2B* have been reported in frontotemporal dementia linked to chromosome 3 (FTD-3) (Urwin et al., 2010). FTD-3 has an onset of 48–67 years and is an autosomal dominant dementia with TDP-43 negative FTLD-U, ubiquitin positive inclusions (Urwin et al., 2010).

As reported in Table 7, several missense mutations are connected with FTD-3 (Skibinski et al., 2005), with familial or sporadic cases of ALS (Parkinson et al., 2006; Urwin et al., 2010), familial frontotemporal lobar degeneration (FTLD) (Ghanim et al., 2010) or CBD (Van der Zee et al., 2008), however only few studies analyzed their pathogenic features in cultured neurons.

**Table 7:** **List of mutations in CHMP2B and their characteristic phenotypes**.

**Subtypes of Dementia**	**Mutation**	**Change in amino acid**	**Type**	**References**
FTD	g.13227A>G	I29V	Unclear	Cannon et al., 2006
FTD	g.18376C>A	T104N	Unclear	Cox et al., 2010
FTD	g.25885A>G	N143S	Unclear	Van der Zee et al., 2007
FTD	g.25899G>T	D148Y	Pathogenic	Skibinski et al., 2005
FTD	g.25950C>T	Q165X	Pathogenic	Van der Zee et al., 2007
FTD	g.26189G>C	p.M178VfsX2/p.M178LfsX30	Pathogenic	Skibinski et al., 2005
FTD	g.26214C>T	R186X	Unclear	Momeni et al., 2006
FTD	g.26218G>A	S187N	Unclear	Ferrari et al., 2010
FTD	g.26276A>C	Q206H	Pathogenic	Parkinson et al., 2006
FTD	c.581C>T	S194L	Unclear	Ghanim et al., 2010

*FTD, Frontotemporal Dementia*.

*All the information reported in the table is derived from a cumulative study of the literature and the database: http://www.molgen.ua.ac.be/ADMutations/default.cfm?MT=0&ML=2&Page=FTD*.

A point mutation has been identified in the 5′ acceptor site of exon 6, causing the production of two abnormal transcripts: CHMP2B^intron5^ with retention of intron 5 and CHMP2B^Δ10^ that has a 10 bp deletion and a sequence frameshift due to the use of a criptic splice site in exon 6 (Skibinski et al., 2005). Both proteins lack 36 amino acids in the C-terminal part with the subsequent absence of VPS4-binding domain and an accumulation of CHMP2B on the endosomal membrane (Skibinski et al., 2005; Urwin et al., 2010). This accumulation suggests that the binding of mutated proteins to the endosomes prevents the recruitment of the correct proteins necessary for the fusion with lysosomes (Metcalf and Isaacs, 2010; Urwin et al., 2010). Indeed large and abnormal endosomal structures are observed in post-mortem brain tissues, in patient fibroblast cultures and in case of overexpression of CHMP2B mutant in PC12 and human neuroblastoma cell lines (Skibinski et al., 2005; Van der Zee et al., 2008; Urwin et al., 2010). Moreover CHMP2B seems to act through a gain of function mechanism in the presence of mutations, since only the transgenic mice expressing CHMP2B^intron5^ show similar neuropathology features observed in FTD-3 patients, whereas the knockout mice with the inactivation of CHMP2B do not show any pathological characteristics (Ghazi-Noori et al., 2012).

Another non-sense mutation replaces a glutamine residue with a stop codon, creating a more severe C-terminal truncation (Van der Zee et al., 2008).

Since CHMP2B is involved in the endosomal trafficking of signal molecules, it could be interesting and possibly relevant for the pathology to check if an altered endosomal process can affect the function of other proteins involved in FTLD, such as progranulin, as is it also suggested by Urwin et al. (2010).

## C9ORF72

### Structure

Large expansions of the non-coding GGGGCC repeat in *C9ORF72* (Chromosome 9 open reading frame 72) were recently demonstrated to cause ALS and FTD (DeJesus-Hernandez et al., 2011; Renton et al., 2011). Indeed 20–80% of familial and 5–15% of sporadic ALS and FTD in North American and European patients show this hexanucleotide expansion with a range of 700–1600 repeats, whereas the normal population carries less than 30 repeats (DeJesus-Hernandez et al., 2011; Renton et al., 2011; Smith et al., 2012). Pathogenic mutations reported in *C9ORF72* are listed in Table 8. *C9ORF72* is localized on chromosome 9 and is composed of 12 exons, with two alternate non-coding first exons (Figure 5C, exon 1a and 1b) (DeJesus-Hernandez et al., 2011). Specifically, the polymorphic hexanucleotides repeat was identified between the two alternatively spliced non-coding exons, through a sequencing approach (DeJesus-Hernandez et al., 2011).

**Table 8:** **List of mutations in *C9ORF72* and their characteristic phenotypes**.

**Subtypes of Dementia**	**Mutation**	**Change in amino acid**	**Type**	**References**
FTD/ALS	g.5321GGGGCC(?)	G4C2 hexanucleotide repeat expansion	Pathogenic	DeJesus-Hernandez et al., 2011
FTD	g.11942A>T	T66S	Pathogenic	Van der Zee et al., 2013

*TD, Frontotemporal Dementia; ALS, Amyotrophic lateral sclerosis*.

*All the information reported in the table is derived from a cumulative study of the literature and the database: http://www.molgen.ua.ac.be/ADMutations/default.cfm?MT=0&ML=2&Page=FTD*.

Depending on alternative transcription initiation, the GGGGCC repeat can be located on the promoter of transcriptional variant 1 or in the intron 1 of transcriptional variants 2 and 3. Variant 2 results from splicing of exons 1a and exons 2–5 whereas variant 3 is composed of exon 1a and exons 2–11.

### Localization and function

Expression array data showed wild type C9ORF72 RNA present in different CNS tissues, including spinal cord and higher expression in the cerebellum (Renton et al., 2011; Table 1).

The protein encoded by *C9ORF72* is still uncharacterized and with unknown function even if it is well-conserved across species (DeJesus-Hernandez et al., 2011).

Recently, Farg et al. (2014) demonstrated for the first time that the endogenous C9ORF72 protein has a function in the regulation of intracellular trafficking processes in the endosomal and autophagy-lysosomal compartments in neuronal cell lines. Therefore, they reported the normal cellular function of C9ORF72 that is essential to understand its role in FTD/ALS.

In particular, they found co-localization in neuronal cell lines and primary cortical neurons of C9ORF72 with four Rab proteins, which are involved in endosomal trafficking. In motor neurons, they found 70% of colocalization with Rab7, which is a fundamental protein implicated in the biogenesis of lysosomes, the transport of endosomes and the maturation of autophagosomes (Gutierrez et al., 2004). A similar mechanism of interaction and recruitment of Rab7 was also described for CHMP2B by Urwin and collaborators. In *CHMP2B* mutant cells, an impaired recruitment of Rab7 onto endosomes was observed with a decreased fusion with lysosomes and a delayed degradation (Urwin et al., 2010).

C9ORF72 protein was also found to colocalize with lysosomes, ubiquilin-2 and autophagosomes, involved in autophagy (Farg et al., 2014). Interestingly, the ability of C9ORF72 to interact with hnRNPs and induce not yet characterized nuclear aggregates and stress granules, could link the C9ORF72 protein with RNA metabolism processes (Farg et al., 2014).

### Implications of RNA in pathogenesis

Immunocytochemistry analysis on human fibloblasts and mouse motor neuron NSC-34 cell line revealed a predominant nuclear localization of C9ORF72 protein (Renton et al., 2011). Immunohistochemical analysis showed C9ORF72 expression in neurons and in FTD- and ALS-affected regions with a predominant cytoplasmic staining and a synaptic localization, but the quantitative mRNA analysis demonstrated that the repeat expansion reduces C9ORF72 transcript variant 1 expression in lymphoblast cell lines of expanded repeats carriers and in frontal cortex samples from unrelated FTLD-TDP patients carrying expanded repeats (DeJesus-Hernandez et al., 2011). The hexanucleotide repetitions present in the C9ORF72 transcript can form G-tetrad units, called G-quartets, where G bases are rearranged in a cyclic pattern with eight hydrogen bonds (Fratta et al., 2012). The presence of RNA G-quadruplexes has been found in different organisms and has been observed *in vitro* and *in vivo* (Kikin et al., 2008; Xu et al., 2010b). Transcripts are enriched in RNA G-quadruplexes structures in the 5′UTR, 3′UTR and in the first exon (Eddy and Maizels, 2008; Huppert et al., 2008). Recently a molecular mechanism was described by which the DNA and RNA G-quadruplexes in C9ORF72 create structures that promote the formation of RNA/DNA hybrids (R-loops) (Haeusler et al., 2014). The pathological mechanism involving*C9ORF*72 gene or its function are not clear, even if several studies showed a decrease in the mRNA levels of some C9ORF72 variants in ALS, which suggests a loss-of-function mechanism (DeJesus-Hernandez et al., 2011; Renton et al., 2011; Gijselinck et al., 2012; Mori et al., 2013b). Moreover, the aberrant transcripts containing the hexanucleotide repeats can accumulate and form structures in the nucleus called RNA foci, which may produce neurodegeneration through a toxic effect (DeJesus-Hernandez et al., 2011). These transcripts can be aberrantly expressed through repeat-associated non-ATG (RAN) translation (Mori et al., 2013b). Several groups reported that the RAN translation of the hexanucleotide repeats produces poly(GP), poly(GA) and poly(GR) proteins, since this type of translation, without an initiation codon, can have all the possible reading frames (Ash et al., 2013; Mori et al., 2013b) and RNA can be also bidirectionally transcribed (Gendron et al., 2013). These RAN proteins can form inclusions in neurons and are considered a hallmark of the disease (Ash et al., 2013; Mori et al., 2013b). The neuronal inclusions can be detected through antibodies that recognize putative GGGGCC repeat RAN-translated peptides, therefore this type of immunoreactivity can be use as a potential biomarker for the disease (Ash et al., 2013).

It is also reported that RNA foci may sequester important RNA binding proteins, causing an alteration inside the cell and a subsequent dysfunction of RNA targets, in a process similar to the formation of RNA foci in myotonic dystrophy type 1 (DM1) (Lee et al., 2013; Mori et al., 2013a; Reddy et al., 2013; Xu et al., 2013). Specifically, one study demonstrated that hnRNP-H is sequestrated by RNA foci, reducing its available amount and its splicing efficiency on different target transcripts (Lee et al., 2013). A recent paper by Gendron et al. (2013) contains detailed descriptions of the proteins found to be sequestered on RNA foci in *in vitro* studies.

The presence of both sense and antisense RNA foci in frontotemporal dementia with the presence of *C9ORF72* repeats (C9FTLD), was demonstrated in patients, specifically in the frontal cortex, motor cortex, hippocampus, cerebellum, and spinal cord (Gendron et al., 2013; Lagier-Tourenne et al., 2013; Mizielinska et al., 2013; Zu et al., 2013). RNA foci were identified in neurons and with lower frequency in astrocytes, microglia, and oligodendrocytes; the highest concentration of foci was found in the frontal cortex region, compared to cerebellum and hippocampus (Mizielinska et al., 2013). However, another work reported that foci are localized with higher frequency in the cerebellum (Lee et al., 2013). Despite this inconsistency, the major part of RNA foci is localized at the very edge of the nucleus, but the explanation for this localization is still unknown (Mizielinska et al., 2013). The cellular toxicity associated with the longer hexanucleotide repeats and the presence of RNA foci was demonstrated using neuroblastoma cells and zebrafish embryos (Lee et al., 2013). One patient, who was homozygous for the *C9ORF72* hexanucleotide repeats, showed a higher proportion of sense and antisence foci with an early onset of FTD and severe pathological characteristics, compared to the heterozygous case (Mizielinska et al., 2013). A recent study found a mechanism for the disease in which the DNA and RNA–DNA structures formed in the repeat regions, alter the RNA transcription, with a result of transcriptional pausing and abortion. The accumulation of abortive transcripts with hexanucleotides repeats, creates G-quadruplexes, and hairpins structures with a binding of essential proteins, leading to nuclear stress, and further defects (Haeusler et al., 2014).

TDP-43 and FUS, two FTD related proteins previously decribed, are structurally related to the hnRNPs that are found to bind C9ORF72 RNA foci (Lee et al., 2013; Mori et al., 2013b), however FUS and TDP-43 do not colocalize with C9ORF72 RNA foci in cells, patient motor neuron cultures or in spinal motor neurons from patients (Lagier-Tourenne et al., 2013; Lee et al., 2013; Sareen et al., 2013). Since TDP-43 is capable to bind through its C-terminal region the hnRNP proteins (Buratti et al., 2005), the accumulation of these ribonucleoprotein on the RNA foci could indirectly influence TDP-43 function, creating a possible link of interaction between these two factors involved in FTD and ALS (Gendron et al., 2013). Indeed most of the cases with *C9ORF72* expansion show TDP-43 inclusions (FTLD-TDP) (DeJesus-Hernandez et al., 2011; Lagier-Tourenne et al., 2013; Mackenzie et al., 2014) with some exception, such as a case in UK with *C9ORF72* repeats with FTLD-tau pathology (Snowden et al., 2012). It was reported that the plasma and CSF level of phosphorylated TDP-43 is significantly higher in patients with FTD carrying *C9ORF72* expansion or *GRN* mutations compared to other FTD patients or healthy controls (Suárez-Calvet et al., 2014). This finding creates another possible link of interaction or regulation between TDP-43 and C9ORF72 that needs further analysis.

## Discussion

In this review we describe the different genes involved in FTD, focusing on their possible interactions, in order to identify a common network of their combined regulations. We created this network focusing on the RNA aspect, an emerging and crucial molecule that plays critical and fundamental functions in the cells. Recently, research has increased its focus onthe role of RNA in neurodegeneration (Renoux and Todd, 2012). We believe that the RNA mediated regulation plays a key role in the unique integration of all the known genes involved in FTD.

**Figure 6:**
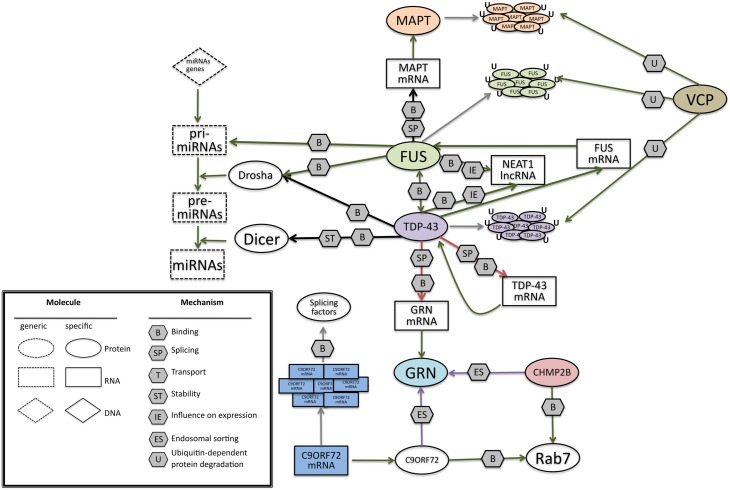
**A possible network of interactions between proteins and RNAs, at the basis of FrontoTemporal Dementia**. Green arrows indicate binding interactions or processes that result in activation or increased expression. Red arrows indicate binding interactions or processes that result in inhibition of activity or reduced expression. Black arrows indicate binding interactions or processes whose result can be either positive or negative. Purple arrows indicate binding interactions or processes which are hypotetical. Symbols as in Legend. lncRNAs, long non-coding RNA; TDP-43, TAR DNA binding protein; FUS, Fused in Sarcoma; GRN, progranulin; MAPT, Microtubule-Associated Protein Tau; VCP, Valosin Containing Protein; C9ORF72. CHMP2B, Charged multivesicular body protein 2b; Rab7, Ras-related protein 7.

In this picture (Figure 6) FUS and TDP-43 RNA binding proteins are at the core of the network, since they often are associated factors that share similar features, with sometimes different but complementary roles (Colombrita et al., 2012). They interact with RNA in three main roles: as RNA binding proteins participating on the different aspects of mRNA processing (Bosco et al., 2010; Colombrita et al., 2011), as regulators of microRNAs processing, and as regulators of lncRNAs. FUS and TDP-43 were both found in aggregates in ALS/FTLD affected neurons (Da Cruz and Cleveland, 2011), nuclear complexes and in cytoplasmic RNPs (Kim et al., 2010b). TDP-43 appears to be the main regulator of this network, being able to interact with FUS pre-mRNA and regulate its splicing, and auto-regulate its own pre-mRNA, causing a reduction of its own expression (Polymenidou et al., 2011).

TDP-43 can also bind GRN pre-mRNA, negatively controlling its splicing and, accordingly, knock-down of TDP-43 was shown to increase the amount of GRN mRNA level (Polymenidou et al., 2011; Colombrita et al., 2012). In the presence of *GRN* mutations, TDP-43 regulation can be altered, causing the formation of inclusions containing TDP-43 (Mackenzie et al., 2006, 2010; Sampathu et al., 2006). Though TDP-43 aggregation is a typical hallmark of many other neurodegenerative disorders, such as Alzheimer's disease, Guam Parkinsonism dementia complex, and Lewy body disease (Dickson, 2008), its impact on FTD in influencing the regulation of the network should not be underestimated.

On the other side, FUS acts as a splicing regulator of MAPT mRNA, indeed it was demonstrated that silencing of FUS alters the splicing of MAPT. In particular, FUS helps the skipping of exon 10 in primary cortical neurons (Ishigaki et al., 2012). The presence and the absence of exon 10 in *MAPT* gene has a fundamental role in the regulation of a delicate balance in the ratio of 4 or 3 repeats that can lead to FTD.

For the recently identified *C9ORF72* gene, large expansions of the non-coding GGGGCC repeat correlate with pathogenesis, making an RNA gain-of function mechanism possible. Indeed the aberrant C9ORF72 transcripts accumulate in nuclear RNA foci and sequester several RNA-binding proteins, including some splicing factors. However, other possible pathogenetic mechanisms are under scrutiny for *C9ORF72*, including impaired transcription of the expanded gene or repeat-associated non-ATG (RAN) translation of expanded transcripts.

In this scenario, two FTD genes code for proteins that fit in the picture not for their relation to RNA, but for their role in protein degradation.

VCP, taking part in the ubiquitin-proteasome pathway and protein turnover (Zhang et al., 2000; Rouiller et al., 2002), could be involved in the degradation of protein inclusions present in different forms of FTD. TDP-43 inclusions were found in the presence of a *VCP* mutation (Neumann et al., 2009a). A direct interaction between VCP and FUS has been observed suggesting a possible convergence in their functions (Wang et al., 2015).

CHMP2B regulates protein trafficking between endosomes and lysosomes and is involved in the protein degradation pathway through lysosomes (Urwin et al., 2010). Therefore, CHMP2B could be relevant for the internalization and transport of neuronal growth factors or signaling molecules such as progranulin.

Recently, a function for the C9ORF72 protein was uncovered, in the regulation of intracellular trafficking processes in the endosomal and autophagy-lysosomal compartments (Farg et al., 2014), providing an additional link to VCP and CHMP2B proteins.

During the preparation of this review a recent study performed by Van der Zee and colleagues have demonstrated ananalysis on a European cohort of 1808 FTLD patients revealing mutationsin SQSTM1 (Sequestosome 1) or p62. The p62 protein is a stress-responsiveubiquitin-binding protein, which is shown to have a role in degradation ofpolyubiquitinated proteins via the proteasome pathway or autophagicprocesses (Van der Zee et al., 2014). This gene was known to be associated with ALS and found as a rare mutation with a frequency of 1–3% in both ALS and FTLD cases. This further intrigued its possible role in pathogenicity with a common patho-mechanism. p62 is present in neuronal and glial ubiquitin-positive inclusions in different tauopathies and synucleinopathies (Van der Zee et al., 2014). The meta-analysis performed by Van der Zee and colleagues revealed that rare mutations clustering in the UBA domain of SQSTM1 may influence disease susceptibility by doubling the risk for FTLD. Further, histopathology analysis of autopsied brain of SQSTM1 mutation carriers demonstrated a widespread of neuronal and glial phospho-TDP-43 pathology. Therefore, this study opens up another target gene SQSTM1, which is known to have implications in FTLD/ALS and additionally associated with TDP-43. Despite further work being needed, to unravel and confirm the details of the proposed network, we foresee that the construction of a picture of the interactions between proteins and RNAs at the basis of the FTD pathology will be of invaluable importance, not only to comprehend the pathogenetic mechanisms but also to develop new and more effective therapeutical approaches. Through the network analysis proposed in this review, it can be foreseen that more genes can be linked to FTD and their roles will possibly fall in to two main categories: regulation of gene expression through RNA or protein degradation. Additionally it could be predicted that novel genes related to FTD in future will be possibly a part of the proposed network.

### Conflict of interest statement

The authors declare that the research was conducted in the absence of any commercial or financial relationships that could be construed as a potential conflict of interest.
